# Mediating Effects of Fear on Mental Health among Undergraduate Students during Open Distance Learning

**DOI:** 10.21315/mjms2023.30.6.10

**Published:** 2023-12-19

**Authors:** Nurhasniza Idham Abu Hasan, Mogana Darshini Ganggayah, Suzainiwati Suhaimi, Nurhasnira Abu Hasan, Nur Faezah Jamal

**Affiliations:** 1 School of Mathematical Sciences Studies, College of Computing, Informatics and Mathematics, Universiti Teknologi MARA, Perak, Malaysia; 2 Department of Econometrics and Business Statistics, School of Business, Monash University Malaysia, Malaysia; 3 BDP Global Services Sdn. Bhd., Menara Takaful Malaysia, Kuala Lumpur, Malaysia; 4 Department of Research, Development and Innovation, Universiti Malaya Medical Centre, Kuala Lumpur, Malaysia

**Keywords:** anxiety, depression, fear, online learning, stress

## Abstract

**Background:**

Online distance learning (ODL) known as the flexible learning environment can trigger a negative impact on students’ mental health. The study aimed to identify the influence of fear as mediator between mental health problem and university students’ perception on ODL.

**Methods:**

This is a cross-sectional study involving a convenient sampling of 258 undergraduate students. Self-administered structured questionnaires adapted from the Depression, Anxiety and Stress Scale-21 (DASS-21) and the Fear of COVID-19 Scale (FCV-19S), were used to assess the severity of psychological symptoms (depression, anxiety and stress) and fear. The perception towards ODL is also designed to assess the students’ perception related to ODL implementation. The data were analysed using descriptive statistics and Structural Equation Modelling-Partial Least Square (SEM-PLS).

**Results:**

Overall, 84.2%, 95.0% and 67.4% of the participants experienced moderate to very severe level of depression, anxiety and stress, respectively. In addition, 82.6% of them suffering with moderate to extreme level of fear, of which 81.8% of participants had a negative view on ODL. The results of SEM-PLS revealed that there are complementary partial mediation effects of fear on the relationship between depression and students’ perception during ODL (*β* = 0.502, *t*-value = 0.828, *P*-value = 0.017). The anxiety (*β* = 0.353, *t*-value = 5.401, *P*-value = 0.000) and stress (*β* = 0.542, *t*-value = 8.433, *P*-value = 0.000) have directly influenced on fear.

**Conclusion:**

This study demonstrated that university students had the prevalence of psychological symptoms and fear during ODL. In line with this, fear contributes significantly to the mental health status of university students and has negatively impacted the students’ perception during ODL implementation.

## Introduction

In recent years, the world has faced a major impact on the lives and livelihoods of people around the world due to the novel coronavirus also known as COVID-19. This pandemic has not only killed many people across the country, but it has also changed many of their cultural practices, including daily lives with no exception in the field of education (1–5). The eighth Malaysian Prime Minister, Tan Sri Dato’ Haji Mahiaddin bin Md. Yasin has announced Movement Control Order (MCO) on 18 March 2020, to control the spread of this pandemic. In the wake of that, both public and private Higher Education Institutions (HIEs) have been urged to close. Alternatively, traditional teaching and learning are switched on to the new norm through online platform, while student performance is assessed through continuous assessment.

Online distance learning (ODL) is a platform that provides students with a flexible learning environment (6). However, due to the sudden appearance of COVID-19, it is not easy to adopt or adapt to online learning without encountering many challenges. Numerous studies show that students faced a variety of difficulties, including a sense of isolation and tension because of their inability to use an online learning platform, boredom, loneliness and a lack of motivation (5–7). All of these challenges have a strong ability to trigger out negative impact on their mental health. Moreover, changes in academic life and education learning environment during COVID-19 are major cause of psycho-emotional disruption, including sleep disturbance and fear, which subsequently increases student suicidal behaviour (8). Although this measure is seen as necessary protective measures, they might have a significant impact on the psychosocial well-being of students.

Amongst mental health issues, depression, anxiety and stress are identified as the most common mental illnesses among students. The unprecedented regulations dramatically altered lifestyles and social relationships and have fuelled a deep level of anxiety that can have devastating impact towards themselves and tends to develop suicidal behaviour. According to the Malaysia Mental Health Association, the prevalence of suicidal ideation either associated with depression or other mental illness was also reported to be the highest among young adults in the aged between 16 years old and 24 years old compared to other age groups categories (9). The latest survey conducted by the Malaysian Department of Mental Health Statistics during the initial lockdown period from 18 March 2020 to 9 June 2020, found that there were 78 suicide attempts in Malaysia, with 1,081 suicide attempts recorded in 2020 alone. A significant increase in suicides also has been reported compared to the previous non-COVID year clearly shows that, the rates of suicide and attempted suicide have had a marked increase since the country’s initial lockdown period (10). The figures are warranting vulnerability to psychological disturbances and infer a probability of academic and psychosocial deterioration on students’ ability to learn and regulate their feelings (11).

Theoretical models state that socioenvironmental stressors direct the pathogenesis of depression (12). Depression is a mood, symptom or disorder in which a person feels sad or loses interest in activities they used to enjoy (13). Consequently, these individuals are more likely to develop stress symptoms in response to stressful situations. Stress is an emotional, physical or mental reaction that causes tension (12). It can occur due to various factors such as social adjustment to a new environment, completing assignments, peer relationships and peer pressure to score high marks in exams (3, 14). A person usually responds to environmental stimuli that are perceived as burdensome or beyond their abilities, which in turn leads to feelings of stress. Stress affects physical, mental and emotional factors that cause physical or mental tension (12). Previous research has found that lack of awareness in dealing with stress leads to anxiety (15).

Anxiety is an internalised arousal of worry that arise from the feelings of insecurity and unpleasantness. Normally, anxiety associated with negative thought and feelings, such as nervousness, lingering apprehension, chronic worry, tension or dread (13). Meanwhile, the psychological and physical symptoms include shivering of hands and lips, dryness in mouth, frequent urination and restless sleep (16). There is evidence in the literature that the anxiety of distance learners stems mainly from their lived experiences and their expectations or assumptions (5, 6, 17). Fear is like anxiety. Nevertheless, fear is an intense physical response to immediate danger that arise from uncertain treat which contradicts to anxiety that arise from ongoing treat (18). The Diagnostic and Statistical Manual of Mental Disorders (DSM) distinguishes anxiety as ‘a future-oriented mood state associated with preparation for possible upcoming negative events.’ Meanwhile, fear is an alarm response to present or imminent danger (real or perceived) (19).

**Figure 1:**
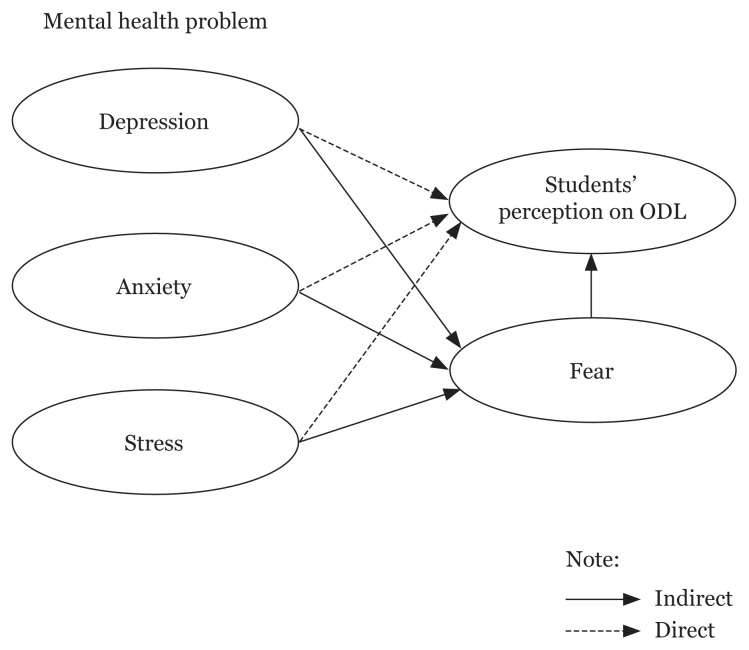
Research model

Although ODL provides a safe place to the students for their learning environment, it can also put them at risk of developing mental health problems. Based on the aforementioned issues, it is clear that the pandemic not only leads to increased mortality from viral infections but also initiates psychological effects among students. In this context, the question that arises is to which level of extent ODL can contribute to the students’ depression, anxiety, stress and fear during the COVID-19 pandemic? Furthermore, in the Malaysian context, the studies on the impact of the COVID-19 pandemic during ODL implementation on the mental health of university students are still limited. In conjunction to that, a study that specifically addresses fear and its’ influence on mental health and students’ perception during ODL is warranted. In view of the issues highlighted above, the aim of this study is to identify the effect of fear as mediator between mental health problem and student’s perception on ODL, thereby filling the gap in the literature that exists in the Malaysian context on the topic under study. Based on the literature, this study proposes a theoretical framework which serves as a proposed structural model as shown in Figure 1.

Figure 1 represents the proposed theoretical research framework on the mediating role of fear between mental health problems and students’ perception on ODL. This research model consists of a direct relationship between independent and dependent variables and an indirect relationship with moderating variables. Although fear is a normal emotion which is facilitating protective behaviours (20), excessive fear may cause severe psychological distress (8). Perceived fear could be a critical factor affecting the students’ emotions during ODL. The findings from this study provide insights to the educators, administrators, and policymakers to enhance ODL implementation that ensures mental and psychological well-being of the students. This study may assist the Ministry of Health (MOH) Malaysia to take actions in increasing the coverage of Mental Health Psychology Support Services (MHPSS) specifically for students in need of support during a pandemic.

## Methods

### Study Design, Participants and Sample

This study was approved by the Institutional Research and Ethics Committee of the university. This cross-sectional study was conducted among undergraduate students at public universities in Malaysia between October 2021 and January 2022. The inclusion criteria for the study were as follows: i) undergraduate students from semester two and above who enrolled as full-time students during the data collection period. Exclusion criteria included: i) the students who dropout from their programme at the time of the study, ii) first-year students since they started their academic course entirely online. The study participation was voluntary and all data were kept confidential.

The sampling method was a convenient snowball sampling with the aim of increasing the number of participants. Initially, the survey invitation with the link to the Google Form were posted via WhatsApp. In the post, each participant who received the link was asked to share with all their friends and relatives. Then, the link was shared with other contacts as well. As proposed in the literature, for Partial Least Squares-Structural Equation Modelling (PLS-SEM), the size of data should be at least 10 times the construct of most question items (21). In the research model, there are seven question items related to the construct of most question items. The minimum number of sample to apply PLS-SEM is 70 (10 × 7 constructs). In total, 258 students participated in this study which meets the minimum sample size requirement.

### Research Instrument

The self-administered structured questionnaire was developed in this study based on an adaptation from previous studies and literature search. The questionnaire comprised of 34 questions, which were divided into four sections. The first section of the questionnaire primarily concerned socio-demographic details of participants: gender, age, education level, living arrangement, ODL preference, area of locality, household income and internet quality.

### Depression, Anxiety and Stress Regarding ODL

The second section was constructed to measure the emotional distress in three major constructs: i) depression which includes common symptoms such as loss of self-esteem, low mood and lack of energy, (ii) anxiety arising from the fear of future negative events and (iii) stress identified by the consistent state of over arousal and low tolerance levels with frustrations in life (13). This scale was adapted from the Depression, Anxiety and Stress Scale-21 (DASS-21) (9) using 21-item and modified specifically to take into account the participants’ emotional distress during online learning environment. All items were rated using the scale of 1–4: 1 = did not apply to me at all; 2 = applied to me to some degree or some of the time; 3 = applied to me to a considerable degree or a good part of the time and 4 = applied to me very much, or most of the time.

DASS-21 is compressed from the original version of the DASS-42 (the long form has 42 items), therefore, the final score of each construct had to multiplied by two (×2). The minimum score is zero and the maximum score is 42. The score of DASS is further categorised into normal, mild, moderate, severe and extremely severe ratings following multiplications of the scores range for each of these subscales. The higher score indicates a higher level of each distress. For depression, categorised by scores as: normal = 0–9 points; mild = 10–13 points; moderate = 14–20 points); severe = 21–27 points and extremely severe = 28–42 points. While, anxiety scores are classified as: normal = 0–7 points; mild = 8–9 points; moderate = 10–14 points; severe = 15–19 points and extremely severe = 20–42 points. The stress scores are classified as: normal = 0–14 points; mild = 15–18 points; moderate = 19–25 points; severe = 26–33 points and extremely severe = 34–42 points. DASS-21 has good validity and reliability. It is widely used in university or college students (1–3, 13, 22–24). In this study, Cronbach’s alpha (CA) was 0.942 for the whole scale and was 0.874, 0.876 and 0.875 for depression, anxiety and stress subscales, respectively.

### Fear during ODL

This scale was adapted and modified from the Fear of COVID-19 Scale (FCV-19S) (25) specifically to take into account the participants’ negative emotional reaction during the online learning environment. The scale consists of six items that relate to fear during ODL. Participants are requested to respond on a five-item Likert-type scale ranging from 1 = strongly disagree to 5 = strongly agree. The total score ranges between 6 and 30, with a high score indicating extreme fear. The overall fear score divides the level of fear into three categories, namely: less = 6–11 points; moderate = 12–22 points and extreme fear = 23–30 points. The instrument presented high reliability and was used widely among university student (26). In the current study, CA was 0.942.

### Students’ Perception on ODL

Perception of ODL was originally developed to assess the students’ perception related to ODL. The development of a questionnaire starts with a literature search using Google Scholar, Science Direct, the National Library of Medicine (PubMed) and Scopus covering students’ perceptions of local and global online learning environments during COVID-19. The questionnaires developed in the previous phase were then reviewed and validated for its’ content by three expert panels consisting of college counsellors, psychiatrists and psychologists. A minimum score of 0.78 for Item-level Content Validity Index (I-CVI) and 0.80 for Scale-level Content Validity Index (S-CVI) were recommended for reproducing content validity (27). The I-CVI and S-CVI from expert review was 1.00 for all items in the construct; therefore, all items were relevant to the respective construct.

Next, face validation was conducted among 13 university students to check for clarity of instructions and language, whether ambiguous or unclear (27). An Item-level Face Validity Index (I-FVI) of 0.78 or higher and Scale-level Face Validity Index (S-FVI) of 0.8 or higher were considered good face validity (27). The result indicates that the value of I-FVI and S-FVI was 1.00. These values show that all items in the questionnaire were understandable and clear for the target participants and was ready for the construct validation process. Finally, the questionnaire was subsequently evaluated for its reliability among 30 students. The acceptable reliability of CA for all construct was greater than 0.70 (28, 29).The results from the pilot study showed the CA coefficient for all constructs was greater than 0.8, indicates high level of internal consistency, and no modifications were needed. Data from pilot evaluations were not included in the final analysis.

The questionnaire in this section consists of seven items in the form of a 5-point Likert scale ranging from 1 = strongly disagree to 5 = strongly agree. The total scores for perception regarding on ODL categorised into negative/positive based on Bloom’s cut-off 80% point out of the total expected score for each part (30). The overall perception of the ODL score was 35 (ranging from 7 to 35); that dichotomised into positive perception of the ODL with total score ≥ 28 and a negative perception of the ODL with a total score < 28. The overall score is between 7 and 35, with higher scores indicating a positive view of ODL. The method of validation is by using pilot study. All items in the questionnaires are presented in the Appendix 1.

### Statistical Analysis

Data were analysed using IBM SPSS version 26.0 and SmartPLS 3.2.7 software. Descriptive statistics were used to describe characteristics of the participants. Results were presented as mean and standard deviation (SD) for continuous variables, while frequency (*n*) and percentage (%) for categorical variables. Next, Structural Equation Modeling (SEM) using SmartPLS was performed to test hypotheses outlined in the conceptual model and to test whether the conceptual model fitted to the data. This analytic approach involved two stages. In the first step, measurement model assessment was used to test the validity and reliability of the measurement model of latent constructs. The second step focused on testing hypotheses about relationships among the variables in the structural model using path analysis. The significance level was set at 0.05 (two-tailed).

### Assessment of the Measurement Model

The measurement model assessment includes individual item reliability and internal consistency. The individual item reliability was tested by factor loading. The internal consistency was tested by latent variable composition reliability (CR) and CA. A factor loading less than 0.5 was considered deleted from the measurement model (31). So that, all factor loadings greater than 0.7 were retained to ensure the model is fit (32). For CR and CA, values ≥ 0.70 was considered acceptable internal consistency (28, 29). The validity refers to the correctness of the scale tool, with the measurement indicators include convergent validity and discriminant validity. For convergent validity, Average Variance Extracted (AVE) ≥ 0.5 indicates acceptable (18). On the other hand, when the correlation among the constructs less than square root of the AVE indicates good discriminant validity (29). Discriminant validity is also to be confirmed if the values of the Hetrotrait-Monotrait (HTMT) ratio of correlations are less than 0.9 (18). A variance inflation factor (VIF) greater than 5 indicates multicollinearity issues (32).

### Assessment of the Structural Model

The validity of the structural model was assessed using the coefficient of determination (R^2^), predictive relevance (Q^2^) and path coefficients (beta values). The R^2^ measured the amount of variance in each dependent variable that explained by independent variables. Hair et al. (32) mentioned that values around 0.67, 0.33 and 0.19 were considered substantial, moderate and weak, respectively. The value of R^2^ ranges from 0 to 1, with larger value indicting more predictive accuracy.

Stone-Geisser’s Q^2^ emerges as the primary metric employed to measure the predictive relevance to assess the research model’s capability to predict (32). Typically, Q^2^ is computed within PLS analysis, utilising an omission distance ranging from 5 in to 10 in (32). The omission distance must be chosen so that the number of observations divided by the omission distance chosen in the model estimation should not be an integer (32). Therefore, omission distance of 9 will be chosen since it does not produce an integer value of the model estimation. Q^2^ was tested by using blind folding procedure with Q^2^ > 0 indicates that the exogenous (independent) constructs have predictive relevance for the endogenous (dependent) construct (32).

## Results

### Socio-Demographic Characteristics of the Respondents

A total of 258 participants were recruited in the study with 218 (84.5%) females and 40 (15.5%) males. There were 206 (79.8%) bachelor’s degrees and 194 (75.2%) in the age group between 21 years old and 23 years old. Over 158 (61.2%) of participants lived in urban areas. Majority of the respondents are living with family (*n* = 181, 70.2%) and their household income less than RM2,500 (*n =* 134, 51.9%). Most students disliked ODL (*n =* 209, 81%). About 152 (58.9%) of the respondents had moderate internet quality, followed by good quality (*n =* 91, 35.3%) and bad quality (*n =* 15, 5.8%). Table 1 shows the summary of the sociodemographic characteristics of the respondents.

Table 2 shows the level of depression, anxiety, stress, fears and perception of ODL among university students. The mean score of depression was 20.791 (SD = 6.932), anxiety was 21.291 (SD = 6.952), stress was 21.174 (SD = 6.934), fear was 18.109 (SD = 6.322) and perception of ODL was 20.889 (SD = 6.386) for all students.

### Measurement Model Analysis

The results of the measurement model assessment are shown in Table 3. Regarding the individual item reliability, outer loadings below 0.50 were deleted. Following this rule of thumb, of the 34 items in our reflective measurement model, only 12 were deleted which are: three items (items 5, 6 and 8) from depression, three items (items 1, 2 and 3) from anxiety, two items (items 1 and 3) from stress, two items (items 5 and 6) from fear and two items (items 1 and 5) from students’ perception on ODL. This procedure was conducted in order to increase the value of CR in the reflective scales. All the remaining items’ factor loadings in each construct were significant and greater than 0.7. Hence, 22 items with loadings between 0.705 and 0.905 were retained.

Internal consistency and construct reliability were good for all constructs, with CA (ranging from 0.871 to 0.893) and CR (ranging from 0.901 to 0.926) which all those values greater than 0.7. In terms of convergent validity, AVE for all constructs was greater than 0.5 (ranged from 0.647 to 0.758) and each construct’s AVE was less than its respective CR, indicating good convergent validity. As regards to multicollinearity, all constructs with their indicators have VIF values below the acceptable threshold. Therefore, no problems related to collinearity were detected.

The discriminant validity was confirmed in the results displayed in Table 4, using two different criteria, the Fornell-Larcker and HTMT criterion. The square root of AVE in the Fornell- Larcker criterion shown to be higher than all other cross-correlations between constructs. In addition, all values for each construct in the HTMT criterion were less than 0.90, indicating good discriminant validity. The measurement model confirms the convergent and discriminant validity. Next, the data were analysed further by examining the model structure.

### Structural Model Analysis

**Figure 2:**
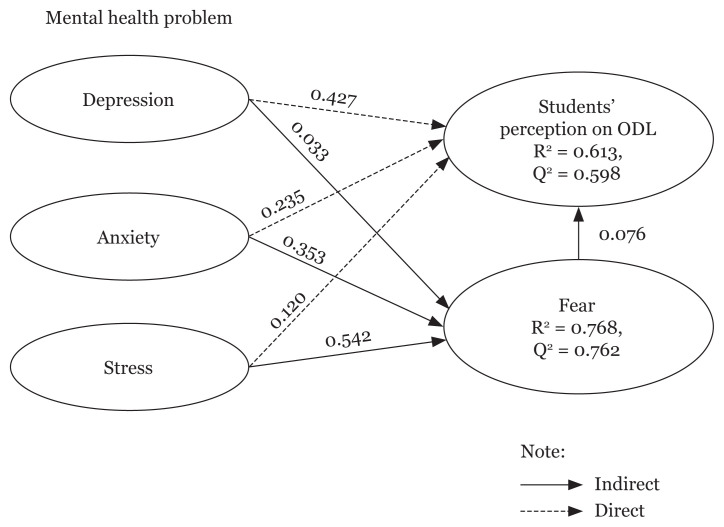
Path model estimation

As proposed in the hypotheses, the predictor variables in this study were depression, anxiety and stress. The criterion variable is students’ perception on ODL and the mediating variable is fear. When the path model was estimated using bootstrapping of 5,000 cases without the interaction of fear as a mediator, the results (Table 5) showed that students’ perception on ODL was significantly influenced by anxiety (*β* = 0.235, *t*-value = 2.565, *P*-value = 0.010) and depression (*β* = 0.427, *t*-value = 6.473, *P*-value = 0.000), except for fear (*β* = 0.076, *t*-value = 0.831, *P*-value = 0.406) and stress (*β* = 0.120, *t*-value = 1.341, *P*-value = 0.180). Notwithstanding that, incorporating fear as a mediator holds significance as it has the potential to elucidate the underlying reasons for the relationship that exists between predictor variables and students’ perceptions of ODL. This inclusion may unveil the authentic relationship between the variables. Examining the predictive power of the model, as displayed in Figure 2, shows that all the predictor variables explained about 61.3% of the variance in the students’ perceptions of ODL. In support of the research finding, Q^2^ for the students’ perception obtained greater than 0 (Q^2^ = 0.598). This demonstrated that the predictive relevance of all predictor variables used in the proposed study.

### Mediating Effects of Fear

Table 5 shows that anxiety (*β* = 0.353, *t*-value = 5.401, *P*-value = 0.000) and stress (*β* = 0.542, *t*-value = 8.433, *P*-value = 0.000) directly influence fear except depression (*β* = 0.033, *t*-value = 0.745, *P*-value = 0.456). However, through fear, there were complementary partial mediation effects on the relationship between depression and students’ perception on ODL (*β* = 0.502, *t*-value = 0.828, *P*-value = 0.017). This is evident from the indirect and direct effects which are all significant and point in the same direction. However, fear did not have any mediation effect between anxiety and students’ perception on ODL (*β* = 0.027, *t*-value = 0.817, *P*-value = 0.091); and between stress and students’ perception on ODL (*β* = 0.041, *t*-value = 0.815, *P*-value = 0.415), indicated by the nonsignificance of indirect effects. In the inclusion of the mediating variable (fear), the predictive power of the model shows more powerful result than without including fear as mediating which explained about 76.8% of variance in assessing the students’ perception on ODL. Therefore, the value of Q^2^ for students’ perception on ODL was significantly higher than without inclusion of fear as mediator (Q^2^ = 0.762). This value demonstrated that inclusion of fear as mediator, provides more predictive relevance of the predictor variables that is explained by the model.

## Discussion

In this study, the prevalence rates of depression, anxiety and stress at moderate to very severe level observed among Malaysian university students were 84.2%, 95.0% and 67.4%, respectively. Meanwhile, 82.6% students suffer moderate to extreme levels of fear, with most of them having a negative perception of online learning (81.8%). Compared with the study conducted in 2020 (1) or even in early 2022 (2) among university students elsewhere in Malaysia, the current findings show that the prevalence rate was high. This result further reinforces most findings that the prevalence in the current findings is much higher than in similar studies conducted pre and even during COVID-19, which is the prevalence of depression, anxiety, and stress that recorded before ranged from 13.9% to 29.3%, 51.5% to 55.0%, and 12.9% to 21.6%, respectively (22). This is consistent with other similar studies in other countries (3, 23, 24).

In a global survey conducted by YoungMinds (33) during the pandemic, 83% of students agreed that the life-threatening COVID-19 was exacerbating their existing mental health conditions due to disrupted learning environment, home isolation and limited social interaction. Not only that, frustration from loss of daily routine could be another reason for these high prevalence rates remained high (34). These prevalence rates are expected to continue to increase and highlight a new attribute associated with the emergence of COVID-19, which is fear. Fear is defined as a negative emotional response resulting from an overestimation of the likelihood of a dangerous situation (35) from an uncertain threat. In the educational setting, fear is presented as a factor that significantly causes psychological distress, which may relate to fears of vagueness and uncertainty. This fear can directly affect directly to the overall learning experience, influencing both the academic performance and well-being of students (36). Throughout the study duration, fear stemmed not solely from concerns related to COVID-19 but also encompassed aspects of the online learning environment, including its infrastructure. Moreover, the limited social interaction during the outbreak further intensified these apprehensions.

In the current finding, it is interesting to note that depression emerges as a factor that influences the students’ perception of ODL. Furthermore, the influences of this depression on the students’ perception of ODL could be exacerbated by the presence of previously unexplored fear, thus constituting a novel contribution. Actions to curb the COVID-19 pandemic, such as home quarantine and remote learning, have been observed to have a profound impact on students’ depression levels. As a result, the isolation of the students from teachers and their classmates destroys social functioning, by increasing their levels of depression which ultimately affects the students’ perception of ODL. Daniel (37) stated that loneliness is one of the main factors causing the student to be depressed. There are evidence of increased loneliness resulting from social isolation which was suspected as a risk factor for depressive symptoms (3). Loneliness affects individuals’ mental condition of being alone and detached (38). Previous studies found that there was a high correlation between loneliness and depression because lonely individuals always have negative perceptions of things which are susceptible to negative emotions (39). Thus, social support from their peers, friends and society, helps to regulate emotions (6, 40).

Interestingly, insights from an online focus group discussion revealed that some students expressed that their primary source of sleep disturbance and mental stress stemmed from heightened apprehension about the uncertainty of their surroundings in academic studies, examinations, the graduation period and job security post-graduation (24, 40). Furthermore, during home learning they were unable to express their fear with peers due to experience fatigue in spending a lot of time on screen, mastering much knowledge in short time, catch up with miss lectures and perpetually accomplishing assignments (4, 7). All of these issues influencing depression. Prolonged fear can directly lead to symptoms of depression or initially results in a state of fear that could later lead to depression. According to the results of this study, the uncertainty and the danger perceived by the undergraduate students can become a fertile breeding ground for fear and finally lead to depression. Lamis et al. (41) also found that students who have a strong social support reported that they do not feel hopeless when they face challenging situations.

The outcomes of this study also indicated that anxiety and stress has been revealed as a factor that influences ODL. While, the effect of anxiety and stress on ODL could not mediated relationship with the existence of fear toward the students’ perception on ODL. This is because fear caused by the uncertainty of direction during the online learning process has nothing to do with anxiety and stress. Even before the pandemic, academic pressures were considered as the main source of stress reported in academic setting including high expectations about academic achievement, competitiveness among peers, heavy workload, financial issues, and strict assignment submission deadlines (14, 41–43). Meanwhile, demanding academic pressure, limited social and personal time during online learning can add layer to the existing stress of life such as lack the essential equipment and facilities to participate online learning, adjusting to a new learning environment, inability for proper time management, unstable connection, new study practices in their home, heavy workload with limited time given from different lecturers (5, 40).

Stress is normal and part of student life. Being a student, it is common to have normal level of stress that drives the student to do their work and duties at university. Moreover, the determination to maintain a flying colour result that usually done in face-to-face causes them unusual stress which also leads to abnormal stress and depression among students and also associated with increased self-injury and suicide attempts (44). Stress has impact on the student’s well-being either physically or emotionally (45). Physically, they frequently suffer headache, fatigue and muscle tightness. Emotionally, the students are frequently suffer from increasing level of frustration and feeling of hopelessness in coping with daily routine. In light of this, fear among student does not mediate the relationship towards the students’ stress in adapting to new learning environment as well as perception in online learning.

It is interesting to realise that a lower stress level does not automatically guarantee an improvement in academic performance among students. However, in such scenarios, students may perceive academic tasks as lacking challenge, potentially experiencing an increased sense of boredom within themselves (46). It is believed that although certain levels of stress may push students toward optimal academic achievement, if stress is not managed effectively due to a lack of coping mechanisms, it can have adverse effects on students. Furthermore, if students are unable to cope with their stressful situations and leave the stressful state untreated, it may result in unfavorable mental health outcomes, increased morbidity, and could potentially have adverse consequences on both their professional and personal lives. The probable explanations could be the students are less tolerant of situations where they are unable to control and cope with the stressful environment (47).

In another situation anxiety can interferes with students’ daily activities. The recent study found that fear was not act as factor that contribute to the student’s anxiety. The sudden switch to online learning leads to an increase in the degree of fear and not link to the anxiety. One of the reasons is due to academic requirements and assignments (3, 40). Despite global concerns, students’ anxieties during online learning are more centered around completing online assignments and related academic requirements. Additionally, on average, students are aware that anxiety arises from performing academic tasks often associated with a heavy workload, and it is not linked to feelings of fear. This concludes that anxiety and stress are closely related to a lack of coping strategies among students (15).

Kiewra (48) stressed out that the perception of the ODL in the instructional process is influenced by an individual’s beliefs about the advantage of distance education for themselves. Those who have had negative experiences of educations in the past and assume that distance learning education may provide the same disempowering learning environment. It is also reported that some university students were concerned about the effectiveness and practicality of online learning as compared with traditional face-to-face learning, which further affects their confidence in online learning (5, 6, 49, 50). Ghaderizefreh and Hoover (50) stated that in order for the students to be satisfied and successful in online learning, they need to motivate themselves and avoid any disturbance while learning. In other words, when they feel motivated, they will be more satisfied with the learning process.

Implication for the study, given that fear negatively affects students’ perception towards online learning and their mental health, hence, faculty should take a more efficient intervention which is focused on the perceived fear during online learning to help these students face these negative emotions. Future research needs to detail how to help students overcome negative emotions, especially fear during online learning. It is important to maintain a well-balanced academic environment to enhance their learning experience. A focus given to the students’ needs and problems can help prevent the harmful effects of negative emotions on their health and academic performance.

## Conclusion

This study reported that most of the university students are seriously affected by mental health problems during ODL. It also demonstrates that fear significantly mediated the relationship between students’ depression and perception towards online learning. In an environment of uncertainty, students often experience academic related fears, such as fear of losing their academic year, fear of delay graduation time and fear of failure. As a result, home isolation with peers during outbreak exacerbated these emotions, by increasing their levels of depression and leading to negative perceptions during online learning.

## Limitation

There are several limitations in this study that need to be acknowledged. Since this study used convenient snowball sampling as the data collection method, the results of this study could not be generalised to all university students in Malaysia. The uneven sampling proportion for various level of semester did not comprehensively evaluate the students’ psychological symptoms throughout their course. These conditions might have influenced the generalisability of the current findings. It is suggested that samples for future research should include students from different degree programs and different semesters to generalise the results to the undergraduate population.

The third limitation is self-reporting as it is subjected to social desirability bias. In addition, response bias might have occurred during data collection. However, for the present study, all the participants available during the data collection period were encouraged to participate in the study and answer honestly, so the bias would be minimised. Furthermore, questions were asked in short form and participants were anonymous so that participants did not have to worry about any negative consequences of participating in the study, thereby minimised bias was caused by self-reporting.

Besides, the questionnaire administration measurements with standard protocols were used to measure students’ mental health and perception during ODL to ensure a minimum source of bias. However, because the researchers had no direct contact with the relevant respondents and were not present when the questionnaires were distributed, there might be a response bias. Given the current pandemic situation, this was the best way to collect data.

## Figures and Tables

**Table 1:** Socio-demographic characteristics of respondents

Characteristics		*N* = 258	%
Gender	Male	40	15.5
	Female	218	84.5
Age (years old)	18–20	46	17.8
	21–23	194	75.2
	24–25	16	6.2
	> 25	2	0.8
Education level	Diploma	52	20.2
	Bachelor’s degree	206	79.8
Living arrangement	Hostel	61	23.6
	With family	181	70.2
	Temporary house	16	6.2
Prefer ODL	Yes	49	18
	No	209	81
Area of locality	Urban	158	61.2
	Rural	100	38.8
Household income (RM)	< 2,500	134	51.9
	2,500–4,849	46	17.8
	4,850–10,959	53	20.5
	10,960–15,039	18	7
	>15,039	7	2.7
Internet quality	Good	91	35.3
	Moderate	152	58.9
	Bad	15	5.8

**Table 2:** Depression, anxiety, stress, fears and perception of ODL

Indicators		Frequency (%)	Mean+ (SD+)
Depression			20.791 (6.932)
	Normal	13 (5.0)	
	Mild	28 (10.9)	
	Moderate	84 (32.6)	
	Severe	83 (32.2)	
	Extremely severe	50 (19.4)	
Anxiety			21.291 (6.952)
	Normal	9 (3.5)	
	Mild	4 (1.6)	
	Moderate	41(15.9)	
	Severe	42(16.3)	
	Extremely severe	162(62.8)	
Stress			21.174 (6.934)
	Normal	52 (20.2)	
	Mild	32 (12.4)	
	Moderate	101 (39.1)	
	Severe	64 (24.8)	
	Extremely severe	162 (3.5)	
Fears			18.109 (6.322)
	Low	45 (17.4)	
	Moderate	141 (54.7)	
	Extremely	72 (27.9)	
Students’ perception on ODL			20.889 (6.386)
	Positive	47 (18.2)	
	Negative	211 (81.8)	

Note:

+ Total scores

**Table 3:** Results of the measurement model assessment

Construct	Item	Factor loading	CA	CR	AVE	VIF
Depression (D)	D1	0.781	0.890	0.919	0.694	1.921
	D2	0.828				2.293
	D3	0.823				2.249
	D4	0.898				3.038
	D7	0.831				2.206
Anxiety (A)	A4	0.866	0.871	0.912	0.722	2.190
	A5	0.884				2.536
	A6	0.860				2.357
	A7	0.784				1.725
Stress (S)	S2	0.841	0.879	0.912	0.677	2.251
	S4	0.801				1.864
	S5	0.882				2.787
	S6	0.871				2.661
	S7	0.705				1.552
Students’ perception on ODL (ODL)	ODL2	0.807	0.863	0.901	0.647	1.929
	ODL3	0.856				2.380
	ODL4	0.729				1.627
	ODL6	0.801				1.874
	ODL7	0.824				2.028
Fear (F)	F1	0.901	0.893	0.926	0.758	2.965
	F2	0.905				3.070
	F3	0.830				2.001
	F4	0.846				2.258

Notes: CA = Cronbach’s alpha; CR = composite reliability; AVE = Average Variance Extracted; VIF = variance inflation factor; There were no interactions and multicollinearity detected

**Table 4:** Discriminant validity analysis

Construct	Fornell-Larcker					HTMT				
	
	A	D	F	ODL	S	A	D	F	ODL	S
A	**0.849**									
D	0.723	**0.833**				0.409				
F	0.809	0.692	**0.871**			0.411	0.766			
ODL	0.701	0.739	0.663	**0.801**		0.599	0.638	0.750		
S	0.796	0.746	0.848	0.690	**0.823**	0.698	0.434	0.747	0.791	

Notes: The bold numbers in the diagonal row represent the square roots of the AVE; HTMT = Heterotrait-Monotrait ratio; A = anxiety; D = depression; S = stress; ODL = students’ perception on ODL; F = fear

**Table 5:** Hypothesis testing of the research model

Relationship	Path coefficients	*t*-statistic	*P*-value	Effect	Decision
A→F	0.353	5.401	0.000*	Direct	Supported
D→F	0.033	0.745	0.456	Direct	Not supported
S→F	0.542	8.433	0.000*	Direct	Supported
F→ODL	0.076	0.831	0.406	Direct	Not significant
A→F→ODL	0.027	0.817	0.091	Indirect	Direct only
A→ODL	0.235	2.565	0.010*	Direct	(No mediation)
D→F→ODL	0.502	8.428	0.017*	Indirect	Complementary
D→ODL	0.427	6.473	0.000*	Direct	(Partial mediation)
S→F→ODL	0.041	0.815	0.415	Indirect	No effect
S→ODL	0.120	1.341	0.180	Direct	(No mediation)

Notes:

* significant at *P* < 0.05;

A = anxiety; D = depression; S = stress; ODL = students’ perception on ODL; F = fear

## Appendix 1

**Instructions**: Please rate the extent to which you agree with the statement below.

1 = did not apply to me at all, 2 = applied to me to some degree or some of the time, 3 = applied to me to a considerable degree or a good part of the time, 4 = applied to me very much, or most of the time[Table t6-10mjms3006_oa]

**Table t6-10mjms3006_oa:**

Item	Questions	Rating			

		1	2	3	4
**Depression**					
D1	I could not seem to experience any positive feeling at all during online learning.				
D2	I found it difficult to work up the initiative to do things due to lack of motivation/support during online class.				
D3	I find myself tired, especially spending too much time in front of a tablet/smart device screen during online learning.				
D4	I felt downhearted during online learning.				
D5	I was unable to become enthusiastic about anything during online learning.				
D6	I felt I was not worth much as a person or as a group member during online learning.				
D7	I felt that life was meaningless since online learning.				
**Anxiety**					
A1	I am worried about not being able to pay tuition fees because my family lost their source of income due to epidemic.				
A2	I am worried of not getting a good result during online classes.				
A3	I am concerned about the time limit to complete all assignments during online learning.				
A4	I was worried about a situation in which I cannot catch up with my studies during online learning.				
A5	I felt I was close to panic when I lost internet connection during online class or final assessment.				
A6	I was worried about not being able to complete the online tasks within the stipulated time allocated by the lecturer.				
A7	I found myself constantly feeling restless without any good reason throughout online learning.				
**Stress**					
S1	I found it hard to wind down in adjusting to a new learning environment.				
S2	I am devastated by the loss of my daily life/study disruption as a student during the pandemic.				
S3	I felt distressed when I lost internet connection during online class or final assessment.				
S4	I found myself getting agitated about heavy workload with limited time given during online learning.				
S5	I found it difficult to relax during online learning.				
S6	I was intolerant of anything that kept me from getting on with what I was doing to achieve high grades during online learning.				
S7	I find it difficult to manage my time while studying online.				

**Instructions**: Please rate the extent to which you agree with the statement below. 1 = Strongly agree, 2 = Agree, 3 = Neutral, 4 = Disagree, 5 = Strongly disagree[Table t7-10mjms3006_oa]

**Table t7-10mjms3006_oa:**

Item	Questions	Rating				

		1	2	3	4	5
**Perception on Open distance learning (ODL)**						
ODL 1	I could not seem to experience any positive feeling at all during online learning.					
ODL 2	I found it difficult to work up the initiative to do things due to lack of motivation/support during online class.					
ODL 3	I find myself tired, especially spending too much time in front of a tablet/smart device screen during online learning.					
ODL 4	I felt downhearted during online learning.					
ODL 5	I was unable to become enthusiastic about anything during online learning.					
ODL 6	I felt I was not worth much as a person or as a group member during online learning.					
ODL 7	I felt that life was meaningless since online learning.					
**Fear**						
F1	I am most afraid of the lack of job opportunities in the future due to COVID-19.					
F2	It makes me uncomfortable to think about graduation time delay due to COVID-19.					
F3	I am afraid of academic failure during online course.					
F4	I am afraid of not meeting the financial requirements of my degree.					
F5	I cannot sleep because I am worried about losing my school year due to COVID-19.					
F6	My heart beat raises when I think about final assessment.					
